# Body composition and mortality in patients undergoing endovascular treatment for peripheral artery disease

**DOI:** 10.1007/s00380-021-01883-2

**Published:** 2021-06-07

**Authors:** Tadashi Itagaki, Soichiro Ebisawa, Kyuhachi Otagiri, Tamon Kato, Takashi Miura, Yusuke Kanzaki, Naoyuki Abe, Daisuke Yokota, Takashi Yanagisawa, Keisuke Senda, Yoshiteru Okina, Tadamasa Wakabayashi, Yushi Oyama, Kenichi Karube, Keisuke Machida, Takahiro Takeuchi, Tatsuya Saigusa, Hirohiko Motoki, Hiroshi Kitabayashi, Koichiro Kuwahara

**Affiliations:** 1 Department of Cardiology, Ina Central Hospital, Ina, Japan; 2 Department of Cardiovascular Medicine, Shinshu University School of Medicine, 3-1-1 Asahi, Matsumoto, 390-8621 Japan; 3 Department of Cardiology, Nagano Municipal Hospital, Nagano, Japan; 4 Department of Cardiology, Shinonoi General Hospital, Nagano, Japan; 5 Department of Cardiology, Nagano Red Cross Hospital, Nagano, Japan; 6 Department of Cardiology, Iida Hospital, Iida, Japan; 7 Department of Cardiology, Saku General Hospital, Saku, Japan; 8 Department of Cardiology, Aizawa Hospital, Matsumoto, Japan; 9 Department of Cardiology, Jōetsu General Hospital, Jōetsu, Japan; 10 Department of Cardiology, Suwa Central Hospital, Chino, Japan; 11 Suwa Red Cross Hospital, Suwa, Japan; 12 Department of Cardiology, Okaya City Hospital, Okaya, Japan

**Keywords:** Peripheral artery disease, Endovascular treatment, Mortality, Body composition

## Abstract

An inverse correlation between body mass index and mortality in patients with peripheral artery disease (PAD) has been reported. However, little information is available regarding the impact of body composition on the clinical outcomes in patients with PAD. This study evaluated the relationships between the lean body mass index (LBMI), body fat % (BF%), and mortality and major amputation rate in patients with PAD. We evaluated 320 patients with PAD after endovascular treatment (EVT) enrolled from August 2015 to July 2016 and divided them into low and high LBMI and BF% groups based on their median values (17.47 kg/m^2^ and 22.07%, respectively). We assessed 3-year mortality and major amputation for the following patient groups: Low LBMI/Low BF%, Low LBMI/High BF%, High LBMI/Low BF%, and High LBMI/High BF%. During the median 3.1-year follow-up period, 70 (21.9%) patients died and 9 (2.9%) patients experienced major amputation. The survival rate was lower in the Low LBMI than in the High LBMI group, and was not significantly different between the Low and High BF% groups. Survival rates were lowest in the Low LBMI/Low BF% group (57.5%) and highest in the High LBMI/High BF% group (94.4%). There were no significant differences in major amputation rate between the Low LBMI and High LBMI groups, and between the Low BF% and High BF% groups. The Low LBMI and Low BF% groups were associated with an increased risk of mortality after adjustment for age, sex, frailty and conventional risk factors [hazard ratio (HR): 4.02; 95% confidence interval (CI) 2.10–7.70; *p* < 0.001 and HR: 4.48; 95% CI 1.58–12.68, *p* = 0.005, respectively], for age, sex, hemodialysis, and prior cerebral cardiovascular disease (HR: 3.63; 95% CI 1.93–6.82; *p* < 0.001 and HR: 4.03; 95% CI 1.43–11.42, *p* = 0.009, respectively) and for age, sex, and laboratory date (HR: 3.97; 95% CI 1.88–8.37; *p* < 0.001 and HR: 3.31; 95% CI 1.15–9.53, *p* = 0.026, respectively). In conclusion, Low LBMI and Low BF% were associated with poor prognosis in patients undergoing EVT for PAD, and mortality was the lowest in the High LBMI/High BF% group compared with other body composition groups.

## Introduction

Peripheral artery disease (PAD) is recognized as a part of systemic atherosclerotic diseases that may impair quality of life by inducing pain while walking and is associated with a poor prognosis [1, 2]. The body mass index (BMI) has reported to be prognostic factors for patients with PAD in several studies [3–6]; however, understanding the BMI is difficult because of the “obesity paradox,” i.e., the inverse correlation between mortality from cardiovascular disease and BMI [3, 7, 8]. From this knowledge, the evaluation of body composition [9, 10], with respect to estimation of fat and fat-free mass, might be important for assessing the long-term outcome of atherosclerotic disease. Indeed, a relationship between the composite of lean body mass (LBM) and body fat (BF) and disease prognosis, especially in patients with coronary heart disease (CHD), has been already reported [11]. However, few studies have assessed the impact of body composition, which is composed of LBM and BF, on the clinical outcomes of patients with PAD. Thus, the evaluation of the composite of LBM and BF is also important to predict the clinical outcomes of patients with PAD. The purpose of this study was to evaluate the impact of the LBM index (LBMI) and BF percentage (BF%) on the 3-year mortality and major amputation rate in patients with PAD who were undergoing endovascular treatment (EVT).

## Materials and methods

This study was a sub-analysis of data from the NAGANO (I-PAD NAGANO) registry, which was a multi-center, prospective, and observational study in Nagano, Japan with the aim of improving disease prognosis in patients undergoing EVT for PAD. All patients with symptomatic PAD who would be undergoing EVT were enrolled. There were no exclusion criteria. From August 2015 to July 2016, 337 consecutive patients from 11 institutes in the Nagano prefecture were enrolled in the I-PAD registry. After obtaining informed consent from all study participants, we recorded their baseline clinical characteristics, including sex, age, comorbidities, medical history, and medications at discharge. Moreover, we performed blood tests, the ankle-brachial index (ABI) test before EVT. Patients were followed prospectively and follow-up data were obtained from hospital charts, directly from patients, or the attending physician 3 years after EVT. Out of 337 patients in the I-PAD registry, 320 patients with enough information for LBMI, BF%, and follow-up data to do the analysis for the study were included in this study. Using the median values for LBMI (17.47 kg/m^2^) and BF% (22.07%) as cutoff values, the 320 enrolled patients were divided into Low and High LBMI and BF% groups. In addition, the patients were categorized into Low LBMI/Low BF%, Low LBMI/High BF%, High LBMI/Low BF% and High LBMI/High BF% groups. The study primary endpoint was all-cause mortality and secondary endpoint was major amputation rate. We statistically assessed the correlation of LBMI and BF% with mortality and major amputation rate. Major amputation was defined as an amputation above the ankle.

The present study was approved by the medical ethics committee of Shinshu University School of Medicine, Japan. As stated above, all patients provided written informed consent before enrollment. The study was registered with the University Hospital Medical Information Network Clinical Trials Registry (UMIN-CTR), as accepted by the International Committee of Medical Journal Editors (No. UMIN000018297). All investigators performed this study in accordance with the Declaration of Helsinki.

Physical and physiological characteristics of each patient were defined as follows: BMI was calculated as body weight (BW) (kg) divided by height squared (m^2^). BW was measured on admission. LBM was calculated using the formula described by Janmahasatian et al.: LBM in males = 9.27 × 10^3^ × BW (kg)/(6.68 × 10^3^ + 216 × BMI); LBM in females = 9.27 × 10^3^ × BW (kg)/(8.78 × 10^3^ + 244 × BMI). The LBMI was calculated as LBM (kg) divided by the height squared (m^2^) [12]. BF was calculated as LBM (kg) subtracted from BW (kg) and BF% was defined as BF (kg) × 100/BW (kg). The Clinical Frailty Scale is a well-established assessment tool of frailty which originated from Dalhousie University in Canada, and ranges from 1 (very fit) to 8 (severely frail) and 9 (terminally ill) [13]. In this study, frailty was defined as a Clinical Frailty score > 4 [13, 14]. Hypertension was defined as systolic blood pressure ≥ 140 mmHg, diastolic blood pressure ≥ 90 mmHg, or ongoing therapy for hypertension. Dyslipidemia was defined as a low-density lipoprotein cholesterol concentration ≥ 140 mg/dL, a high-density lipoprotein cholesterol concentration < 40 mg/dL, a triglyceride concentration ≥ 150 mg/dL, a previous diagnosis of dyslipidemia, or current treatment with lipid lowering agents. Diabetes mellitus was defined as a hemoglobin A1c level ≥ 6.5%, fasting plasma glucose ≥ 126 mg/dL, 2 h plasma glucose level ≥ 200 mg/dL after a 75 g oral glucose tolerance test, non-fasting plasma glucose ≥ 200 mg/dL, a previous diagnosis of diabetes mellitus, or treatment with oral hypoglycemic agents or insulin injections. Critical limb ischemia (CLI) was defined as Rutherford category 4, 5, or 6 [15]. The estimated glomerular filtration rate (eGFR) was calculated using the following formula: for males, eGFR (mL/min/1.73m^2^) = 194 × creatinine ^−1.094^ × age^−0.287^; for females, eGFR = 194 × creatinine ^−1.094^ × age^−0.287^ × 0.739 [16].

Continuous variables were expressed as means ± standard deviation if normally distributed and as medians with interquartile ranges if other than normal. Categorical variables were presented as numbers and %. Continuous variables were compared using the unpaired Student’s *t* test or one-way analysis of variance if normally distributed, and the Mann–Whitney *U* test or the Kruskal–Wallis test was used for non-normal distributions. Comparisons of categorical variables were conducted using Fisher’s exact tests. The survival rates were calculated using the Kaplan–Meier method and compared by the log-rank test. Cox proportional hazards regression analysis was performed to evaluate the prognostic significance of LBMI and BF%. Multivariate analysis was performed by multiple models to adjust for the effects of baseline risk factors for mortality. LBMI and BF% were adjusted for potential confounders without strong correlation with other variables. Model 1 was adjusted for age, sex, frailty, current smoking, hypertension and diabetes mellitus. Model 2 was adjusted age, sex, hemodialysis, prior stroke, prior myocardial infarction and prior heart failure hospitalization. Model 3 was adjusted for age, sex and laboratory data (levels of hemoglobin, albumin, eGFR and C-reactive protein). A *p* value < 0.05 was considered statistically significant. All statistical analyses were performed with EZR (Saitama Medical Center, Jichi Medical University, Saitama, Japan), which is a graphical user interface for R (The R Foundation for Statistical Computing, Vienna, Austria) [17].

## Results

The baseline characteristics of the patients are listed in Table 1. During the median 1135 day (3.1 year) follow-up period (interquartile range 1.8–3.4 years) for the 320 patients, all-cause death was observed in 70 patients (21.9%). Follow-up rates at 1 year, 2 years and 3 years were 95.6%, 88.8% and 79.7%, respectively. The values for BMI and LBMI in non-survivors were significantly lower than those in survivors. The value of BF% tended to be lower in non-survivors than in survivors, but there was no significant difference between the two groups. The % of female and frail patients was greater in the non-survivor than in the survivor groups. The non-survivor group contained a higher % of patients with certain comorbidities and CLI compared to the survivor group. Non-survivors had a lower ABI of the index limb than survivors. The laboratory tests showed that non-survivors had lower levels of hemoglobin, albumin, and eGFR and higher levels of C-reactive protein and B-type natriuretic peptide than survivors.

**Table 1:** Baseline characteristics of the study population

	Overall (*n* = 320)	Survivors (*n* = 250)	Non-survivors (*n* = 70)	*p* value
Age (years)	73.5 ± 9.0	72.6 ± 9.1	76.4 ± 8.3	0.002
Female, *n* (%)	83 (25.9)	58 (23.2)	25 (35.7)	0.044
BMI (kg/m^2^)	22.1 [20.1, 24.6]	22.8 [20.8, 25.1]	20.3 [18.5, 22.2]	< 0.001
LBMI (kg/m^2^)	17.5 [15.3, 18.7]	17.8 [16.1, 19.0]	15.9 [14.1, 17.4]	< 0.001
BF%	22.1 [18.0, 28.5]	22.5 [18.7, 27.2]	19.5 [15.0, 31.4]	0.063
Frailty (CFS ≥ 5), *n* (%)	82 (25.6)	46 (18.4)	36 (51.4)	< 0.001
Current smoker, *n* (%)	41 (12.8)	36 (14.9)	5 (7.6)	0.153
Comorbidities				
Hypertension, *n* (%)	265 (80.0)	208 (83.2)	55 (78.6)	0.38
Dyslipidemia, *n* (%)	194 (60.6)	161 (64.4)	32 (45.7)	0.006
Diabetes mellitus, *n* (%)	165 (51.6)	122 (49.0)	43 (61.4)	0.079
Hemodialysis, *n* (%)	76 (23.8)	45 (18.0)	30 (42.9)	< 0.001
Prior stroke, *n* (%)	65 (20.3)	51 (20.4)	14 (20.0)	1.00
Prior myocardial infarction, *n* (%)	56 (17.5)	36 (14.4)	20 (28.6)	0.012
Prior HF hospitalization,* n* (%)	44 (13.8)	23 (9.2)	21 (30.0)	< 0.001
Atrial fibrillation, *n* (%)	59 (18.4)	40 (16.0)	18 (25.7)	0.078
Lower limb and procedure characteristics				
ABI of index limb	0.64 [0.55, 0.76]	0.65 [0.56, 0.79]	0.58 [0.49, 0.66]	< 0.001
Critical limb ischemia, *n* (%)	114 (35.6)	69 (27.6)	45 (64.3)	< 0.001
TASC-II classification C/D, *n* (%)	148 (46.2)	110 (44.0)	38 (54.3)	0.137
Stent placement, *n* (%)	251 (78.4)	200 (80.0)	51 (72.9)	0.25
Number of stents	1 [1, 2]	1 [1, 2]	1 [0, 2]	0.179
Laboratory data				
Hemoglobin (g/dL)	13.0 [11.5, 14.5]	13.6 [11.9, 14.7]	11.3 [10.7, 12.4]	< 0.001
Albumin (g/dL)	3.9 [3.5, 4.2]	4.0 [3.7, 4.3]	3.5 [3.1, 3.7]	< 0.001
eGFR (mL/min/1.73m^2^)	49.0 [18.1, 64.1]	53.0 [33.8, 66.1]	23.0 [33.8, 55.0]	< 0.001
Total cholesterol (mg/dL)	170.0 [147.0, 192.3]	173.0 [148.5, 193.0]	159.0 [136.0, 184.0]	0.015
HDL cholesterol (mg/dL)	48.0 [41.0, 58.0]	50.0 [42.0, 59.0]	44.5 [39.0, 55.8]	0.045
LDL cholesterol (mg/dL)	93.5 [75.0, 115.8]	95.0 [78.0, 115.0]	89.5 [71.0, 117.0]	0.133
Triglycerides (mg/dL)	109.0[79.0, 160.5]	113.0 [82.0, 167.5]	89.0 [66.5, 132.5]	0.003
Hemoglobin A1c (%)	6.2 [5.8, 7.0]	6.2 [5.7, 7.0]	6.3 [5.8, 7.1]	0.69
C-reactive protein (mg/dL)	0.17 [0.05, 0.60]	0.15 [0.05, 0.45]	0.44 [0.11, 2.09]	< 0.001
BNP (pg/mL)	91.6 [33.3, 208.7]	69.8 [29.1, 150.0]	316.5 [109.2, 612.7]	< 0.001
Medications				
Aspirin, *n* (%)	258 (80.1)	204 (81.6)	52 (75.4)	0.31
Thienopyridine, *n* (%)	236 (73.3)	183 (73.2)	51 (72.9)	1.00
Cilostazol, *n* (%)	85 (26.4)	64 (25.6)	21 (30.0)	0.45
Statins, *n* (%)	170 (53.1)	146 (58.4)	24 (34.3)	< 0.001
ACEIs and/ or ARBs, *n* (%)	174 (54.4)	141 (56.4)	33 (47.1)	0.177
Beta-blockers, *n* (%)	92 (28.6)	64 (25.6)	26 (37.1)	0.071
Warfarin, *n* (%)	34 (10.6)	23 (9.2)	10 (14.3)	0.26
Direct oral anticoagulants, *n* (%)	27 (8.4)	21 (8.4)	5 (7.1)	1.00

Values are means ± standard deviation, medians (interquartile range), or *n* (%)

*ABI* ankle-brachial index, *ACEI* angiotensin-converting enzyme inhibitor, *ARB* angiotensin-receptor blocker, *BF%* body fat percentage, *BMI* body mass index, *BNP* B-type natriuretic peptide, *CFS* clinical frailty scale, *eGFR* estimated glomerular filtration rate, *HDL* high-density lipoprotein, *HF* heart failure, *LBMI* lean body mass index, *LDL* low-density lipoprotein, *TASC* Trans-Atlantic Inter-Society Consensus for the Management of peripheral arterial disease

Table 2 shows the baseline characteristics for the 4 patient groups that were based on high and low LBMI and BF%. There were significant differences between the groups for age, frailty, smoking status, dyslipidemia, prior myocardial infarction and HF hospitalization, ABI, CLI, levels of hemoglobin and albumin, high-density lipoprotein cholesterol, triglycerides, and B-type natriuretic peptide.

**Table 2:** Baseline characteristics of the study population classified by high and low LBMI and BF%

	Low LBMI/Low BF% (*n* = 78)	Low LBMI/High BF% (*n* = 82)	High LBMI/Low BF% (*n* = 82)	High LBMI/High BF% (*n* = 82)	*p* value
Age (years)	74.5 ± 8.4	76.0 ± 10.0	72.3 ± 8.4	70.9 ± 8.5	0.001
Female, *n* (%)	0 (0.0)	82 (100.0)	0 (0.0)	1(1.3)	< 0.001
BMI (kg/m^2^)	19.3 [17.9, 20.4]	21.4 [19.2, 23.4]	22.6 [22.0, 23.3]	26.0 [25.1, 27.0]	< 0.001
LBMI (kg/m^2^)	16.5 [15.7, 17.1]	14.2 [13.2, 15.0]	18.1 [17.8, 18.4]	19.6 [19.2, 20.0]	< 0.001
BF%	14.5 [12.0, 16.4]	33.8 [31.1, 36.0]	19.8 [18.9, 20.8]	24.6 [23.4, 26.0]	< 0.001
Frailty (CFS ≥ 5), *n* (%)	25 (32.1)	29 (35.4)	16 (19.5)	12 (15.4)	0.008
Current smoker, *n* (%)	17 (23.3)	5 (6.2)	13 (16.5)	6 (8.0)	0.007
Comorbidities					
Hypertension, *n* (%)	58 (74.4)	68 (82.9)	69 (84.1)	68 (87.2)	0.20
Dyslipidemia, *n* (%)	37 (47.4)	45 (54.9)	53 (64.6)	58 (74.4)	0.003
Diabetes mellitus, *n* (%)	36 (46.1)	39 (47.6)	42 (51.2)	48 (61.5)	0.22
Hemodialysis, *n* (%)	19 (24.4)	24 (29.3)	17 (20.7)	15 (19.2)	0.45
Prior stroke, *n* (%)	17 (21.8)	13 (15.9)	15 (18.3)	20 (25.6)	0.44
Prior myocardial infarction, *n* (%)	15 (19.2)	5 (6.1)	20 (24.4)	16 (20.5)	0.007
Prior HF hospitalization, *n* (%)	19 (24.4)	12 (14.6)	7 (8.5)	6 (7.7)	0.010
Atrial fibrillation, *n* (%)	11 (14.1)	18 (22.0)	15 (18.3)	14 (17.9)	0.65
Lower limb and procedure characteristics					
ABI of index limb	0.61 [0.49, 0.71]	0.63 [0.53, 0.74]	0.68 [0.57, 0.78]	0.68 [0.58, 0.80]	0.007
Critical limb ischemia, *n* (%)	34 (43.6)	39 (47.6)	20 (24.4)	21 (26.9)	0.002
TASC-II classification C/D, *n* (%)	45 (57.7)	38 (46.3)	31 (37.8)	34 (43.6)	0.084
Stent placement, *n* (%)	62 (79.5)	59 (72.0)	66 (80.5)	64 (82.1)	0.42
Number of stents	1 [1, 2]	1 [0, 2]	1 [1, 2]	1 [1, 2]	0.53
Laboratory data					
Hemoglobin (g/dL)	12.9 [11.5, 14.4]	11.8 [10.5, 13.2]	13.3 [11.8, 14.6]	14.4 [12.2, 15.0]	< 0.001
Albumin (g/dL)	3.8 [3.5, 4.2]	3.7 [3.3, 4.1]	3.9 [3.6, 4.2]	4.0 [3.8, 4.4]	< 0.001
eGFR (mL/min/1.73m^2^)	53.0 [17.0, 73.0]	43.0 [14.5, 60.5]	48.0 [21.7, 63.5]	55.0 [37.0, 63.4]	0.43
Total cholesterol (mg/dL)	170.8 ± 35.9	178.6 ± 35.9	166.6 ± 33.8	168.2 ± 32.7	0.159
HDL cholesterol (mg/dL)	49.0 [42.0, 55.0]	54.0 [44.5, 63.0]	46.0 [40.0, 54.5]	46.0[39.0, 56.5]	0.012
LDL cholesterol (mg/dL)	92.5 [75.0, 114.5]	98.5 [80.0, 122.3]	92.5 [73.8, 116.3]	92.0 [77.0, 110.0]	0.56
Triglycerides (mg/dL)	103.0 [76.0, 156.8]	102.0 [66.8, 141.3]	104.0 [81.0, 153.0]	137.0 [85.0, 175.0]	0.013
Hemoglobin A1c (%)	6.2 [5.8, 6.9]	6.1 [5.8, 6.9]	6.3 [5.7, 6.9]	6.5 [5.9, 7.1]	0.20
C-reactive protein (mg/dL)	0.18 [0.05, 1.08]	0.20 [0.05, 0.88]	0.17 [0.06, 0.48]	0.16 [0.06, 0.33]	0.39
BNP (pg/mL)	133.6 [37.5, 434.0]	98.2 [57.9, 211.8]	96.9 [35.3, 244.8]	52.4 [22.8, 119.5]	0.004
Medications					
Aspirin, *n* (%)	62 (82.7)	62 (75.6)	65 (77.4)	67 (84.8)	0.44
Thienopyridine, *n* (%)	59 (78.7)	55 (67.1)	60 (71.4)	60 (75.9)	0.53
Cilostazol, *n* (%)	20 (26.7)	22 (26.8)	25 (29.8)	18 (22.8)	0.78
Statins, *n* (%)	29 (38.7)	41 (50.0)	47 (56.0)	53 (67.1)	0.003
ACEIs and/ or ARBs, *n* (%)	30 (40.0)	44 (53.7)	50 (59.5)	50 (63.3)	0.028
Beta-blockers, *n* (%)	22 (29.3)	21 (25.6)	26 (31.0)	21 (26.6)	0.90
Warfarin, *n* (%)	6 (8.0)	16 (19.5)	2 (2.4)	9 (11.4)	0.003
Direct oral anticoagulants, *n* (%)	5 (6.7)	6 (7.3)	9 (10.7)	6 (7.6)	0.95

Values are mean ± standard deviation, median (interquartile range), or *n* (%)

*ABI* ankle-brachial index, *ACEI* angiotensin-converting enzyme inhibitor, *ARB* angiotensin-receptor blocker, *BF%* body fat percentage, *BMI* body mass index, *BNP* B-type natriuretic peptide, *CFS* clinical frailty scale, *eGFR* estimated glomerular filtration rate, *HDL* high-density lipoprotein, *HF* heart failure, *LBMI* lean body mass index, *LDL* low-density lipoprotein, *TASC* Trans-Atlantic Inter-Society Consensus for the Management of peripheral arterial disease

**Fig. 1:**
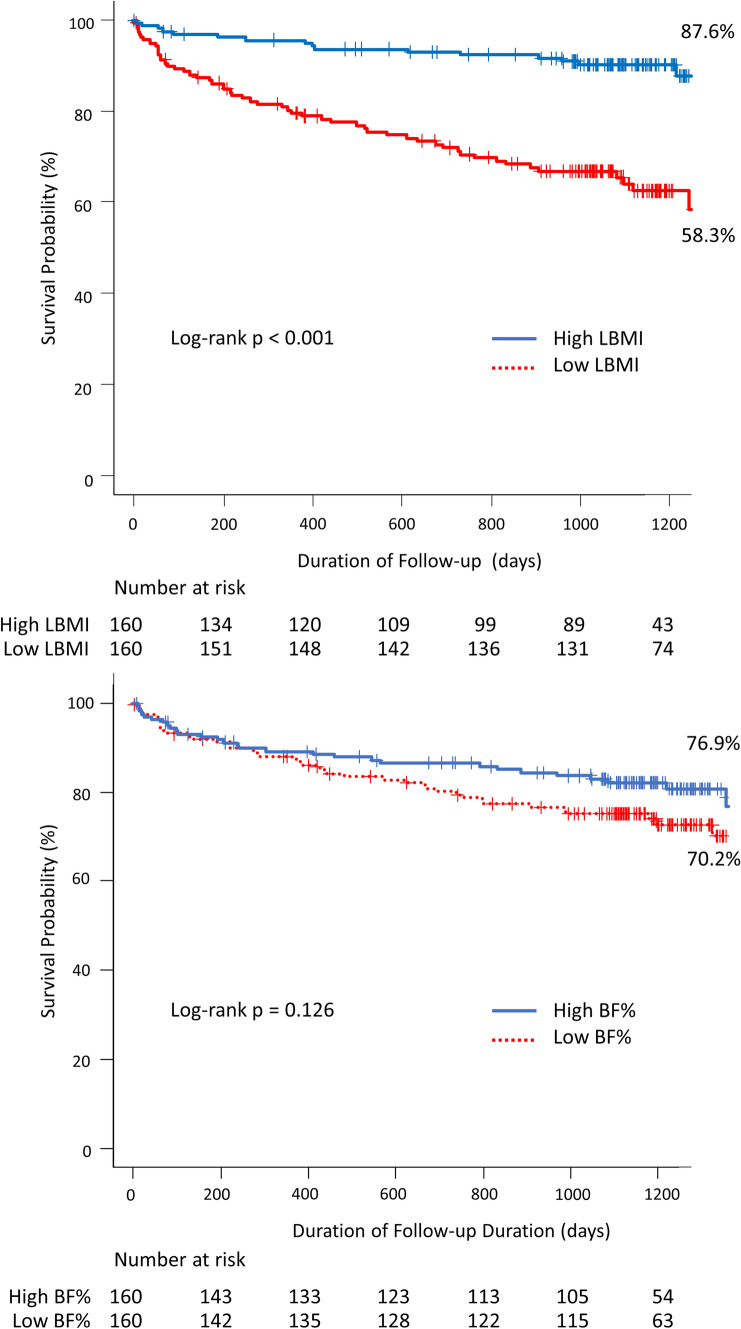
Kaplan–Meier survival curves of patients with PAD stratified by the LBMI and BF%. **a** Kaplan–Meier curves for probability of survival according to the LBMI. The probability of survival was lower for patients in the Low LBMI group than in the High LBMI group. **b** Kaplan–Meier curves for probability of survival according to BF%. There was no difference in the probability of survival between patients in the Low BF% and High BF% groups. BF% body fat %; LBMI lean body mass index; PAD peripheral artery disease

Kaplan–Meier analysis indicated that the probability of survival was lower for patients in the Low LBMI group than in the High LBMI group (Fig. 1a). There was no difference in the probability of survival between patients in the Low BF% and High BF% groups (Fig. 1b).

**Fig. 2:**
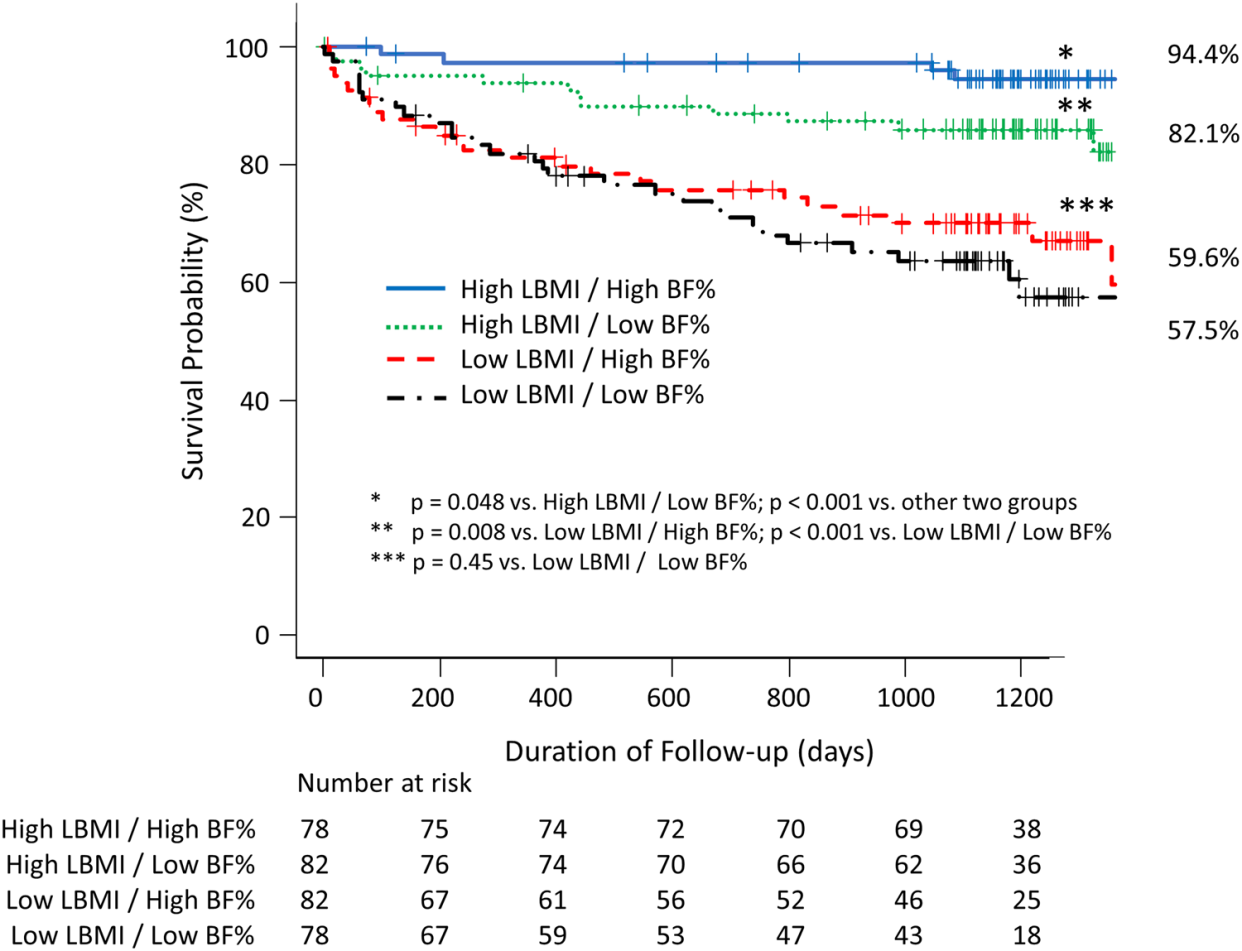
Kaplan–Meier survival curve of patients categorized by combinations of High and Low LBMI and BF%. The probability of survival was significantly highest in the High LBMI/High BF% group of the 4 groups. BF% body fat %; LBMI lean body mass index

Figure 2 shows the survival curves for the 4 LBMI/BF combination groups. Probability of survival was significantly highest in the High LBMI/High BF% group of the 4 groups. Patients in the High LBMI/Low BF% group had a higher probability of survival than in both Low LBMI groups. There was no significant difference in survival between the Low LBMI/ High BF% and Low LBMI/ Low BF% groups.

**Fig. 3:**
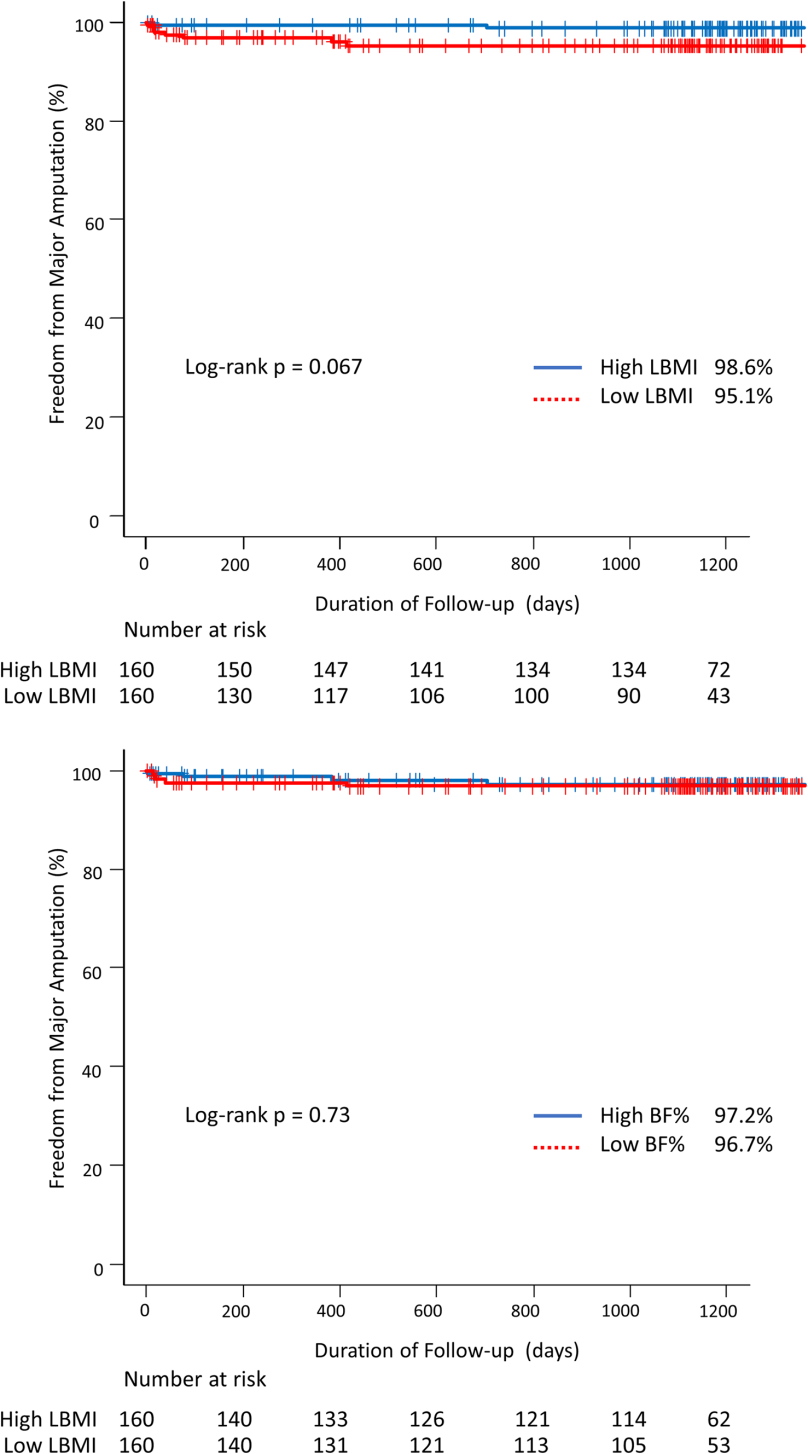
Kaplan–Meier curves for major amputation of patients with PAD stratified by the LBMI and BF%. **a** Kaplan–Meier curves for major amputation according to the LBMI. The freedom from major amputation was not differ significantly between Low LBMI and High LBMI groups. **b** Kaplan–Meier curves for major amputation according to the BF%. The freedom from major amputation was not differ significantly between Low BF% and High BF% groups. BF% body fat %; LBMI lean body mass index; PAD peripheral artery disease

**Fig. 4:**
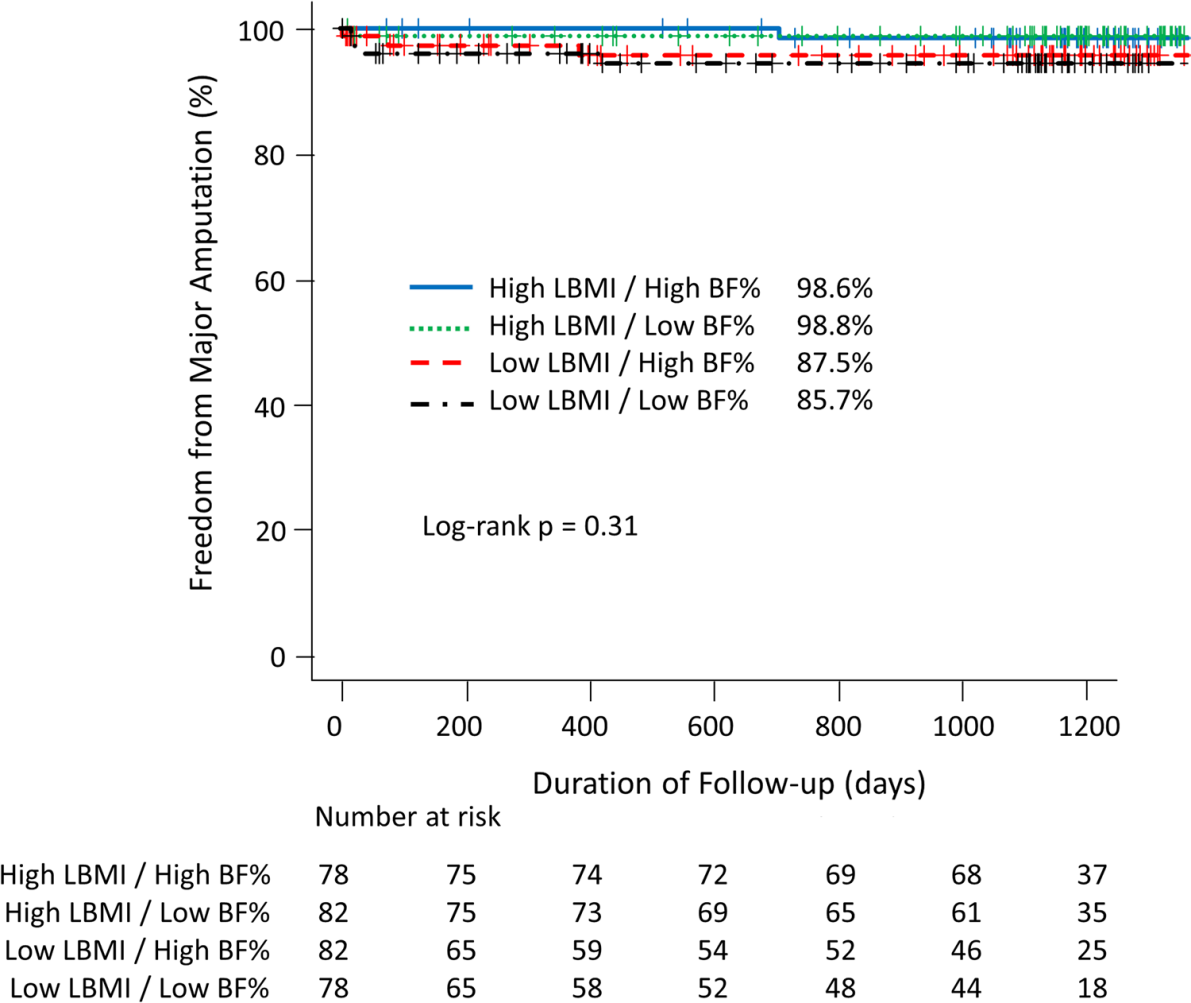
Kaplan–Meier curve for major amputation of patients categorized by combinations of High and Low LBMI and BF%. There was no significant difference in the freedom from major amputation among the 4 groups. BF% body fat %; LBMI lean body mass index

There was no significant difference in freedom from major amputation rate between patients in the Low LBMI and High LBMI groups (Fig. 3a), that was similar between patients in the Low BF% and the High BF% (Fig. 3b). Similarly, there was no significant difference in freedom from major amputation among the 4 LBMI/BF% combination groups (Fig. 4).

Table 3 shows the multivariate Cox proportional hazards analysis for all enrolled patients. Both low LBMI and low BF% predicted mortality after adjustment for age, sex, frailty, current smoking, hypertension and diabetes mellitus. Similarly, in the model 2 adjusted for age, sex, hemodialysis, prior stroke, prior myocardial infarction and prior heart failure hospitalization, and model 3 adjusted for age, sex, and levels of hemoglobin, albumin, eGFR and C-reactive protein, both low LBMI and low BF% also predicted mortality. In addition, the same analysis was performed for patients with claudication (except CLI) (Table 4). In the patients with claudication, a low LBMI was also an independent risk factor for mortality in the all models. Although a low BF% did not predict mortality in the model 3, a low BF% increased the risk of mortality.

**Table 3:** Univariate and multivariate Cox proportional hazards analysis for all enrolled patients

	Low LBMI		Low BF%	
	HR (95% CI)	*p* value	HR (95% CI)	*p* value
Univariate analysis	4.15 (2.37–7.26)	< 0.001	1.45 (0.90–2.33)	0.128
Model 1	4.02 (2.10–7.70)	< 0.001	4.48 (1.58–12.68)	0.005
Model 2	3.63 (1.93–6.82)	< 0.001	4.03 (1.43–11.42)	0.009
Model 3	3.97 (1.88–8.37)	< 0.001	3.31 (1.15–9.53)	0.026

Model 1 is adjusted for age, sex, frailty, current smoking, hypertension and diabetes mellitus. Model 2 includes age, sex, hemodialysis, prior stroke, prior myocardial infarction and prior heart failure hospitalization. Model 3 includes age, sex, and levels of hemoglobin, albumin, estimated glomerular filtration rate and C-reactive protein

*BF* body fat, *CI* confidence interval, *HR* hazard ratio, *LBMI* lean body mass index

**Table 4:** Univariate and multivariate Cox proportional hazards analysis in patients with claudication alone

	Low LBMI		Low BF%	
	HR (95% CI)	*p* value	HR (95% CI)	*p* value
Univariate analysis	3.37 (1.45–7.83)	0.005	4.08 (1.53–10.88)	0.005
Model 1	8.23 (2.84–23.90)	< 0.001	10.21 (1.33–78.23)	0.025
Model 2	5.33 (2.11–13.49)	< 0.001	8.52 (1.12–64.79)	0.039
Model 3	6.58 (1.92–22.57)	0.003	5.56 (0.71–43.44)	0.101

Model 1 is adjusted for age, sex, frailty, current smoking, hypertension and diabetes mellitus. Model 2 includes age, sex, hemodialysis, prior stroke, prior myocardial infarction and prior heart failure hospitalization. Model 3 includes age, sex, and levels of hemoglobin, albumin, estimated glomerular filtration rate and C-reactive protein

*BF* body fat, *CI* confidence interval, *HR* hazard ratio, *LBMI* lean body mass index

## Discussion

In this study, we have shown that a low LBMI and BF% are positively associated with mortality in patients who underwent EVT for PAD. A low LBMI was associated with survival of PAD patients; however, the association for BF% was limited. When assessing combinations of LBMI and BF%, the High LBMI/High BF% and Low LBMI/Low BF% groups had the least and highest mortality, respectively. In addition, a low LBMI and BF% were significantly associated with mortality after adjustment for confounders. Similar results were observed in patients with claudication (except CLI). In contrast, major amputation rates were not different significantly according to the value of LBMI and BF%.

A retrospective cohort study reported the prognostic significance of LBMI and BF in patients with stable CHD [11]. In this report, both low LBMI and BF were independent risk factors for a poor prognosis, and the survival rate was lowest in the Low LBMI/Low BF group and highest in the High LBMI/High BF group. Other studies demonstrated that lower BF [18] and a lower BF category (Gallagher classification) after adjustment for age and sex [19] were independent risk factors for higher mortality in patients with stable CHD. Furthermore, in a recent report from a cardiovascular disease cohort that included CHD, cerebrovascular disease, hypertension, and heart failure, decreased LBM and BF were associated with increased mortality, although the protective role of muscle mass was emphasized [20]. Our results for patients with PAD are similar to the above studies [11, 18–20].

This study showed that compared to a low LBMI, a high LBMI was associated with lower mortality in PAD patients. This is biologically plausible. Skeletal muscle mass has been reported as an important factor for improving arteriosclerosis. A loss of muscle mass exacerbates arteriosclerosis, which may lead to a poor outcome for PAD patients [21–24]. Moreover, a lower LBM was associated with frailty and decreased physical activity, both of which are major risk factors of mortality from cardiovascular disease and in the general population [25–27].

This study found that higher BF% was correlated with better survival in patients with PAD. However, these findings do not necessarily mean that more BF equates to a better prognosis for PAD. Because population in this study consisted of Japanese patients with relatively low BF %, it remains unclear excess BF leads to better prognosis. The mechanism for the protective role of BF in PAD is unclear, but there are several hypotheses that may provide an explanation [28, 29]. Additionally, because of the prevalence of obesity-related diseases, obese patients may take more care to prevent these diseases and receive earlier and more frequent medical care.

This study did not show significant association between major amputation rate and body composition. However, the number of events was small and was not significant enough to assess the association. Therefore, evaluation about the association in a larger cohort will be needed.

The strongest point of our results was the novel finding that assessment of the combinations of LBMI and BF% in patients with PAD was useful for predicting prognosis. This result was also observed in patients with CLI. Long-term prognosis for PAD patients still requires improvement. Devices, drugs, and overall strategies for PAD treatment have limitations. Therefore, risk stratification based on physical information, such as the assessment of LBMI, BF%, and their combination, and the establishment of a therapeutic strategy based on such information may be an important means to improve the prognosis of PAD.

There were several limitations in our study. (1) The sample size was relatively small and the follow-up period was relatively short. (2) Only patients with PAD undergoing EVT were selected, so the clinical results of PAD patients who did not have EVT were not included. (3) The number of enrolled female patients was relatively small compared to males. However, generally, the number of female patients with PAD is small [1, 30, 31]. (4) LBMI and BF% vary depending on the time on measurement, because BW in patients with hemodialysis and heart failure easily changed. However, because the sample size was not large enough, we could not analyze excluding patients with hemodialysis and heart failure. (5) The lack of follow-up data for LBMI and BF%.

In conclusion, our findings suggested that both low LBMI and low BF% were associated with a poor prognosis in patients with PAD undergoing EVT. Mortality was the highest in the Low LBMI/High BF% and Low LBMI/Low BF% groups and lowest in the High LBMI/High BF% group. Further studies in larger population are needed to validate our findings in general populations and to find a favorable body composition to improve the prognosis in patients with PAD.
